# Attention: a descriptive taxonomy

**DOI:** 10.1007/s40656-022-00538-3

**Published:** 2022-11-15

**Authors:** Antonios Kaldas

**Affiliations:** Department of Philosophy, Macquarie University, Sydney, NSW Australia

**Keywords:** Attention, Taxonomy, Definition, gorilla experiment.

## Abstract

The term *attention* has been used to mean so many different things that some have despaired of it being useful at all. This paper is devoted to bringing a modicum of order to the chaos through the time-honored device of categorization. The chief purpose of this paper is to introduce a comprehensive descriptive taxonomy of the nuanced ways the term *attention* may be employed. It is presented in table form, followed by elucidations and illustrations of each of its items. But first, I offer reasons why a taxonomy of attention is needed and explore some of its possible benefits. After presenting the taxonomy, I sketch by way of example how it might be applied to two interesting questions: is the umbrella term *attention* still useful?; and, what is it that ties the subdefinitions of attention together yet distinguishes them from other kinds of cognition?

## Introduction

When I use a word,’ Humpty Dumpty said, in rather a scornful tone, ‘it means just what I choose it to mean — neither more nor less.

The question is,’ said Alice, ‘whether you **can** make words mean so many different things.

‘The question is,’ said Humpty Dumpty, ‘which is to be master — that’s all.’ … ‘When I make a word do a lot of work like that,’ said Humpty Dumpty, ‘I always pay it extra.’ (Carroll, 1887, Chap. 6)

The term *attention* has been made to work harder than most words in the English language. Attention has been considered to be anything from too obvious to require definition (“Everyone knows what attention is” (James, 1890))^1^ to impossible to define (“No one knows what attention is” (Hommel et al., 2019)). What is unquestionable is that attention is definitionally promiscuous—the term has been used in a vast variety of ways. Yet few would disagree that attention lies at the very heart of human experience, if not human existence. There is a long history of reflection upon the concept (Mole, 2017; Styles, 1997, Chap. 2) and today, it not only figures in disciplines as varied as biology, philosophy of mind, cognitive science, sociology, information technology, and literary studies; it is increasingly becoming a valuable economic resource. The rapid advance in the sophistication and ubiquity of electronic communication has seen competition for the attention of consumers reach levels unimaginable to past generations, with the potential for dire consequences for human flourishing (e.g., Turkle, 2008; Williams, 2018).

Given both the ubiquity and importance of attention, it would be nice if authors and thinkers, both within and across these disciplines had a common language for communicating what it is more exactly they mean when they use the term. That is what I attempt here, as a first pass liable to future improvement.

In Sect. 2 I explore the value of having a comprehensive taxonomy of attention and. Section 3 is the main part of the paper. Here I outline the basic principles that underlie the compilation of the taxonomy and draw some common distinctions about kinds of attention that cut across my taxonomic categories. I then present my taxonomy, first in table form, followed by explanations and examples of each term. In Sect. 4, I illustrate the usefulness of the taxonomy by sketching how it might be applied to two interesting questions: is the umbrella term *attention* still useful?; and, what is it that ties the subdefinitions of attention together yet distinguishes them from other kinds of cognition?

## The value of a comprehensive taxonomy

The central purpose of this paper is to develop and present a comprehensive taxonomy of the different uses of the cognitive term *attention* in the academic literature. It is closely related to the approach biologists take to grouping and taxonomizing living creatures, and for many of the same reasons. Consider the modern taxonomy of life. It first arose as a way of classifying different lifeforms phylogenetically—according to their physical features. Later, with the advent of Darwinian evolution, it came to be realized that increasing complexity of form could be mapped onto patterns of evolution: form signifying development.

My taxonomy of attention is based more or less on “form”—the obvious essential defining features for each entry. The entries are then organized into levels and groups according to their similarity, just as for the taxonomy of life. We can then consider these relationships and ask (as we did for the taxonomy of life) why these particular groupings? What is it about the entries that causes them to fall neatly into this pattern and not another? How do different groups relate to each other? And what is it that determines whether something belongs in a taxonomy of attention or not? It is unlikely that so neat an answer as the evolution of life will explain the structure of the taxonomy of attention, but it is an interesting question to explore.

The taxonomy of life provides inquirers working on different parts of the taxonomy with a *lingua franca* that facilitates clear communication between them. Similarly, each researcher can more precisely define (and communicate to others) exactly which aspects of *attention* she is researching by locating it within the taxonomy. What is more, a researcher investigating, for example, *attention-as-vigilance* can use the taxonomy to better define how her work relates to the work of others on, say, *attention-as-amplification*. The taxonomy provides a framework that—like the taxonomy of life—can be explored on many different levels: neurochemical; neuronal; anatomical; physiological; psychological; experiential; social; etc. Further, it provides a *lingua franca* that can be useful not only to workers within a discipline, but also across the broad variety of disciplines that are interested in attention and facilitate communication between them.

The taxonomy of life does not directly answer the question of just what *life* is, and neither does my taxonomy answer the question of just what *attention* is. But in both cases, having the varieties of the entity in question logically arranged may facilitate reflection on what it is that ties them all together and at the same time distinguishes them from other entities (life from non-life; attention from non-attention). This is a deep and difficult question that lies beyond the scope of this paper, but I offer some brief thoughts in Sect. 4.

One can hardly imagine modern biology without the Linnaean taxonomy of life. But in the field of cognition the value of taxonomies is also evident in—to name a few examples—Aristotle’s catalogue of virtues (Aristotle, 2011), the Big Five model of personality (McCrae & Costa, 2003), and the taxonomies of memory (Michaelian & Sutton, 2017, Sect. 2) and cognitive artifacts (Heersmink, 2013). Yet no such satisfactory taxonomy that is comprehensive enough to perform the kinds of functions the taxonomy of life performs currently exists for attention.^2^

… the field and literature lack a common language to communicate and connect their work with each other. Drawing analogy with another folk story, to abandon the term attention would cause all blind men or women to feel different parts of the elephant, not realizing that they are touching the same animal. (Chun et al., 2011, p. 91)

The fact that we may not realize we are touching different parts of the elephant by no means suggests that the very concept of *elephant* is too unwieldy to be of any use. In fact, it is only by collecting and cataloguing those different data that the community of the blind can come to understand just what an elephant in all its glory is, and how their different conceptions of it fit together. That is one goal for my taxonomy of attention (see Sect. 4).

## The taxonomy

In this section I build a detailed taxonomy of definitions of attention, based on conceptual distinctions, and illustrated and explained by examples from the existing attention literature. As Ruff observes, attention is not a single, monolithic mechanism or process, and encompasses more than mere biology.

… it appears that decades of intensive research have resulted in a body of work that may allow us to formally define attention from a mechanistic neural perspective. Such developments are encouraging, and may ultimately help us to understand which subdefinitions of the concept of attention may be justified by biological reality. Time will tell whether such notions will venture outside of neuroscience, to complement classic introspective definitions of attention based on verbal descriptions and mental states. (Ruff, 2011, p. 18)

To develop this taxonomy, I adopted the methodology of the biologists and of Samuel Johnson (1709–1784), whose pioneering work captured the contemporaneous use of language in eighteenth century England in the remarkably influential *A Dictionary of the English Language* (1755). His method consisted chiefly of gathering and collating examples of the many different usages of a word from existing literature on slips of paper (Boswell, 1917, anno 1747), which he then defined, illustrated with the examples he had gathered, and organised alphabetically. The only difference is that I have organised my entries conceptually rather than alphabetically. That is, I have sought to group together usages of the word *attention* that share essential features. These groups proved amenable to being organised into a hierarchical structure, much like the tree of life (although unlike a dictionary). The samples collected come chiefly from the scientific literature on the philosophical and psychological study of attention. There may be other uses of attention in, say, literary or medical literature that might perhaps add to or modify the current taxonomy.

While a definition ought ideally to capture the essence of that which it defines, ultimately, it is a tool intended for a certain purpose. Different approaches to defining a concept may better suit different purposes. Purpose is not the sole arbiter of the validity of a definition, of course. Stipulation can be taken too far. But nature rarely imposes extremely narrow restrictions on how it allows itself to be carved up. “Scientific terms are not given in nature. They are worked out, often over generations of data collection and debate” (Baars, 1997, p. 363). In this paper I take neither a *commonsense* nor a *stipulative* approach (de Brigard & Prinz, 2010), but a *descriptive* approach, identifying and categorizing some of the most interesting and common phenomena that have been thought to belong under the umbrella of *attention*.^3^ This descriptive approach does not require that any of the definitions be considered as *sine qua non* of attention. Like biological taxonomies, my attentional taxonomy is not set in stone, but liable to improvement; and it is not the only way to carve up attention, although it reflects real conceptual distinctions. It might be objected that approaching attention via the taxonomy is too restrictive and prevents other, more imaginative approaches. But this objection only bites if the taxonomic approach were put forward as the *only* approach, or even the *chief* approach. My objectives are far more modest—to add the taxonomy to the existing approaches as a supplement rather than a supplanter.

The three chief categories of my taxonomy are *Behavioral, Phenomenal*, and *Mechanistic*. These represent three quite distinct conceptions of how one might characterize *attention*. *Behavioral* conceptions characterize attention by the objective and measurable behaviors of the person as a whole; *Phenomenal* conceptions characterize attention by the subjective character of experience; and *Mechanistic* conceptions characterize attention by the objective subpersonal mechanisms involved in producing both the objective behavior and the subjective experience of attention. The *Mechanistic* class is by far the richest, and I have employed Bechtel’s useful classification of *Parts, Operations*, and *Organization* as the framework for logically arranging groups of entities here.

The richness of the variety of uses of the term *attention* extends beyond my taxonomy; in “other dimensions” so to speak. The taxonomy is to be used as one tool of classification, then, among others, much as color is just one way of characterizing an object among others (shape, size, location, etc.). For example, the entries in my taxonomy are not limited to any one sensory modality, or even to sensory perception itself, but may apply across all of human cognition, or indeed, non-human cognition.^4^ It might be argued that some of these definitions do not define attention *per se*, but rather either conditions for, or consequences of, attention. But my task here is chiefly descriptive—cataloguing actual uses of the term that may serve to guide further discussions on such questions.

Further, there are a number of distinctions commonly employed in the literature (Table 1) that cut across the categories of my taxonomy and might also be considered other tools, or other dimensions on which any given instance of attention can be plotted (the taxonomy being just one dimension). For example, one taxonomical subcategory of attention is *salience*. *Attention-as-salience* may be instantiated in different ways, each of which plots a point upon the axes of the distinctions in Table 1. It may, for example, be either an internal or an external target that is salient, or salience may be almost exclusively involuntary rather than voluntary, and so on. But the thing that makes it *salience* and not any other kind of attention is that essence or phenomenal concept of “standing out,” of seeming important.

**Table 1:** A summary of some important distinctions among kinds of attention

*Where is attention focused?*	Internal Attention Attention to internal states	External Attention Attention to the external world
*How is attention recruited?*	**Endogenous / ** **Top-Down Attention** Attention is directed by the subject	**Exogenous / ** **Bottom-Up Attention** Attention is drawn by external stimuli
*Is volition involved?*	**Voluntary Attention** I choose to attend	**Involuntary Attention** My attention is drawn without my choice
*Are there degrees of attention?*	**Focal Attention** The “center of attention”	**Background Attention** Partial attention to the periphery of what is attended
*What is being attended?*	**Spatial Attention** Attention to a region or locus in (for example) a visual field	**Object Attention** Attention to a composite object as a whole **Feature Attention** Attention to specific features of an object, rather than the object as a whole
*How is attention related to time?*	**Synchronic Attention** Attention at a moment in time	**Diachronic Attention** Attention over or through time
*At what level is the attention?*	**Personal-Level Attention** The subject attends	**Sub-Personal-Level Attention** Some subsystem or cognitive process attends

All of these other “dimensions” may be employed to further refine the resolution of any description of a given instance of attention. Of course, they need not all be used all the time. One must choose the relevant tools for the purpose at hand. But it is nice to have a full toolbox.

Within the taxonomy of attention itself, any given instance of the term *attention* is more likely to encompass more than one of these definitions. Unlike the taxonomy of life, here, hybrids are the rule, not the exception. Thus, it might be that the mechanism of *attention-as-salience* is *attention-as-abundance*—it is when the brain devotes rich processing resources to the perception of a target that the subject feels it is salient. The taxonomy also provides levels of resolution. For some purposes, it may be sufficient to define attention simply as *selection*, while for others, it may be helpful to distinguish *selection-as-spotlight* from *selection-as-competition*. Further, many of the entries overlap conceptually. Some form of selection seems to be inherent in many of the entries in the taxonomy. Thus, there are other plausible schemas that might be developed that use different principles for organizing the entries and their groupings and hierarchy. For these reasons, my taxonomy is liable to revision and improvement. Even if the overall schema is not convincing to some, some of its distinctions may still prove useful.

I present the Taxonomy in table form first (Table 2) to give the reader an overview, and then proceed to “show my working” (and provide some context) by explaining each item and illustrating it with citations to actual usage. To provide a common thread, I also use the example of the *invisible gorilla*. In the famous *inattentional blindness* experiment of Simons and Chabris (1999); subjects watch a video of basketball players passing the ball to each other, and are asked to count the passes. Many subjects do not notice that a man in a gorilla suit walked right through the middle of the scene while they were counting. References to entries in the taxonomy table will henceforth be identified by an italicized title plus a numerical code from the table in square brackets, e.g., *Expectation* [3.B.II.d]. I leave out the numerical code when an entry is repeated in the same passage.

**Table 2:** Taxonomy of Definitions of Attention

1. Behaviorist	A. Behaviorism		
	B. Behavioral Markers		
2. Phenomenal	A. Chief Tenancy of Consciousness		
	B. Confidence		
	C. Clarity / Vividness		
	D. Salience		
	E. Fixedness		
	F. Directedness		
3. Mechanistic	A. Parts	I. Anatomical Regions	
		II. Connectomes	
	B. Operations	I. Access	a. Simple Access (but not accessibility)
			b. Abundance
			c. Increase / Amplification
			d. Maintenance
			e. Influence / Effects
		II. Detection / Recognition	a. Orienting
			b. Vigilance / Monitoring
			c. Scanning / Searching
			d. Expectation
			e. Alerting
			f. Tracking
		III. Selection	a. Filtering
			b. Gateway
			c. Spotlight
			d. Zoom In / Out
			e. Exclusion / Suppression / Absorption / Dedication / Absence of Distraction
			f. Competition
			g. Selection for Action
		IV. Control	
		V. Coherence	
	C. Organization	I. Cyclic Pathways	
		II. Feedback Loops	
		III. Contextual Interconnection	

### Behaviorist definitions

Behaviorism is, roughly, the positivist view that behavior can be explained via reference solely to external (and objective) factors to the exclusion of any need to refer to the inner workings of the mind or the experience of the subject (Graham, 2019, Sect. 1). *Behaviorist* [1.A] definitions of attention, then, characterize attention in purely external, objective, observable behavioral terms. Today, Behaviorism has fallen somewhat out of favor (Graham, 2019, Sect. 7), but there have been authors who formulated a definition of attention in *Behaviorist* terms. For example, B. F. Skinner (1953, pp. 123–124) considers attention to be “a controlling *relation*—the relation between a response and a discriminative stimulus … the criterion is whether the stimulus is exerting any effect upon our behavior.”

In the nineteenth century (before the advent of the Behaviorist movement) Ribot (1890, Sect. 19)^5^ considered the outward marks of attention to be “the necessary conditions, the constituent elements, the indispensable factors of attention.” This intuition is a strong one and there is something to be said for the importance of the consequences of attention as being part of that which defines it. In practice, that is the most common way we recognize attention in others, and occasionally, even in ourselves. Take the invisible gorilla example. We identify the absence of attention in the test subjects by the fact that they are unable to verbally report the presence of the gorilla, and perhaps by observing that their eyes track the passes but never fixate on the gorilla—all of which are behavioral markers. So, a distinction between *Behavioral Markers* [1.B] of attention, and *Behaviorist* [1.A] definitions of attention is one worth making. While the exclusionary principles of Behaviorism that leave out the “inner workings” are untenable today, *Behavioral Markers* continue to play an important role in attention research, as when, for example, attention is defined as occurring just when a subject meets certain observable experimental criteria, or passes a certain threshold on an experimental scale of attention.

### Phenomenal definitions

*Behaviorism* leaves out something important. Often when we attend, there is something it is like to attend (Nagel, 1974). But is attention *always* phenomenal? On some definitions, it is. What I am calling *Phenomenal* [2] definitions of attention are those approaches that include a phenomenal aspect of attention as a *sine qua non* feature of attention: there is *always* something that it is like to attend.^6^ In terms of phenomenal experience, *Phenomenal* attention is the antithesis of *Behaviorist* attention—it grounds attention in the very phenomenality that Behaviorism ignores. I have grouped *Phenomenal* definitions in six sub-categories: *chief tenancy of consciousness; confidence; clarity and vividness; salience; fixedness;* and *directedness*.^7^

#### “Chief Tenancy of Consciousness”

That which is attended is just that of which we are most conscious, the *Chief Tenancy of Consciousness* [2.A]. Whatever happens to predominate in one’s current conscious cognition is said to be attended. This concept of attention is found in the work of B.F. Bradley and J.S. Mill (de Brigard & Prinz, 2010, p. 52). Thus, the subjects cannot report the presence of the gorilla because its image never dominated their phenomenal content, and they can only report that which does so dominate.

Defining concepts such as utilizing an *Abundance* of cognitive resources [3.B.I.b] and being in the *Spotlight* of attention [3.B.III.c] are similar to this subdefinition but are merely functional definitions and not essentially phenomenal. There might be nothing *it is like* to execute them; they can occur subconsciously or even unconsciously.

#### Confidence

The definition of attention as *Confidence* [2.B] can be traced back at least as far as Descartes:

So long as we attend to a truth which we perceive very clearly, we cannot doubt it. But when, as often happens, we are not attending to any truth in this way, then even though we remember that we have previously perceived many things clearly, nevertheless there will be nothing which we may not justly doubt so long as we do not know that whatever we clearly perceive is true. (Descartes, 1988, p. 309)

Whatever we experience without attending is relatively vague and unsure. Paying attention to a thing brings a greater degree of certainty to the experience of that thing, and this is reflected as the phenomenal sense of confidence. Mole concludes that for Descartes, “the move from radical doubt to certainty about the truth of particular clear and distinct ideas—is, therefore, a transition that is mediated by attention” (Mole, 2017, Sect. 1.1).

The subjects in the invisible gorilla experiment did not report the gorilla because they failed to attend to it in a way that would have given them a confidence about its passing through the scene. Interestingly, Simon and Chabris (1999) tried to tease out a report of the gorilla from subjects using not one question, but a series of four, gradually more explicit questions, starting with “did you notice anything unusual?” and ending with “did you see a gorilla walk across the screen?” (p. 1068). Yet only one out of nearly two hundred subjects showed any kind of wavering through this series of questions. That is, nearly all the subjects were quite confident in their answers, whether yes or no, regardless of the suggestive line of questioning. On this definition of attention, this *Confidence* tracks perfectly where the subjects’ attention was directed, whether to the gorilla, or not.^8^

#### Clarity and vividness

While *Confidence* is about a propositional certainty *that* something is thus and not otherwise, there is a closely related *Phenomenal* definition of attention that defines it as a perceptual *Clarity* or *Vividness* [2.C]. In practice, it is hard to imagine vividly seeing a bright red rose and not being confident that you see it, but the two can come apart at least conceptually. Without doubt, attention usually enhances clarity and vividness. The vague and shadowy figure I might have felt flitting around my visual field becomes a *Clear* and *Vivid* (if somewhat unconvincing) gorilla the moment I turn my attention upon it. Even if I look upon a visual scene through the out-of-focus lens of a camera, I can experience the very lack of proper focus clearly and vividly if I attend to it.^9^

This idea of attention as *Clarity* seems to have attracted interest in the early twentieth century,^10^ but has fallen largely out of favor in modern times. Treisman (1964, p. 12) laments that the idea of attention as “the increased clearness of a particular idea” has proven to be sterile in psychological research, Watzl (2011a, pp. 151–152) argues against the very similar perceptual “determinacy view.” But the idea has continued to play a role in at least some research. For example, Baddeley and Andrade (2000) showed that selectively taxing the working memory of a sensory modality attenuated the “phenomenological vividness” of perceptions in that modality. And Schlagbauer et al., (2018) found that contextual cueing—a way to draw a subject’s attention to the configuration of visual display elements—enhances the “clarity” (p. 2) of the subjective experience of both the target object and the surrounding visual configuration.

However, there is some reason to doubt that attention alone is the sole determinant of *Phenomenal Clarity and Vividness*. For example, Wassell et al. (2015) found that higher blood (and salivary) progesterone concentration correlates with an enhancement of the vividness of voluntary visual mental imagery, which is perhaps in keeping with other research that suggests that females tend to have more vivid imagery than males (Campos & Pérez, 1988). However, the mechanism of the enhancement has yet to be elucidated. The possibility remains that elevated levels of progesterone might exert their enhancing effect upon *Vividness* via enhancing attention, as adrenaline (epinephrine) most likely does in a classic fight or flight response.

#### Salience

*Salience* [2.D] has been described as an “attend to me” signal (Sawaki & Luck, 2010). Potential targets of attention are more *Salient* just insofar as they are more likely to draw attention. *Salience* is generally characterized as bottom-up attention (Table 1)—features that “stand out” are more *Salient*. But in fact, it is likely to be a complex interplay between bottom-up standing out and top-down context-sensitive biasing (Egeth et al., 2010, p. 130).^11^ Whereas a gorilla in a basketball game would normally be highly *Salient*, the top-down attention to following the basketball overpowers this bottom-up signal. As a *Phenomenal* definition of attention, the *Salience* is about that phenomenal sense of urgency or importance that—whether bottom-up or top-down—draws attention to a target. In the positive symptoms of schizophrenia, this sense ectopically results from unimportant stimuli (Fletcher & Frith, 2009).

Ruff (2011, p. 5) observes that *Salience* is readily capable of being characterized in the non-phenomenal terms of neural processing patterns: a bottom-up effect of one or more stimuli evoking a stronger neural response than other stimuli competing for limited neural response resources (compare *Selection*, [3.B.III]). Indeed, much of the attentive processing of *Salience* occurs subconsciously, and it is possible for unconscious objects—masked nudes, for example—to attract attention-as-salience (Jiang et al., 2006). This intuition is further supported by work such as that of van Swinderen (2005, p. 324) which suggests that *Salience* mechanisms might operate in producing attention in fruit-flies, whose capacity for phenomenal consciousness remains an open question (Barron & Klein, 2016; Key et al., 2016; Tiffin, 2016).

#### Fixedness

*Fixedness* [2.E] is the experience of being restricted to a narrow train of content such that one is unable to escape that train and enter into other trains. The subjects’ task of counting the passes fixes their attention narrowly on the ball and the players, excluding interpretations of the scene that include an ectopic gorilla. Baars (1988, pp. 143–145) develops this view of attention by considering a sentence like the following:

The ship sailed past the harbor sank.

Most readers will initially have trouble making sense of this sentence, until they realize it may be read as, “the ship—which was sailed past the harbor by someone—later sank.” Before this realization, the reader’s interpretation of the sentence is *Fixed* by the assumption that the most obvious subject of the verb “sailed” is the ship, rather than the people sailing the ship. Like top-down imperatives in *Salience*, context is an important dimension of *Fixedness* here for Baars. Attention-as-fixedness is having our experience trapped by powerful hierarchies of context in a particular way of experiencing to the exclusion of other possible ways of experiencing. Thus, *Fixedness* is an inherently *Phenomenal* form of attention. For example, “in absorbed states of mind—in reading an engrossing novel or watching an entrancing motion picture—we are deaf and blind to the world” (p. 145).^12^

#### Directedness

Related to Baars’ fixedness, the idea of *intentionality* (Jacob, 2019) has also been conscripted to the task of defining attention. Here, one attends to a thing just when one *Directs* [2.F] their thoughts to that thing. For example, understanding a word might be thought intuitively to imply both attending to that word and being conscious of the word and its meaning. The subjects’ attention (and therefore the content of their consciousness) is constituted by their directing their thought towards the movement of the basketball, but never to the gorilla. Posner (1994, pp. 7400–7402) discusses attention as “attending to ideas.” Some more recent accounts that might fit plausibly under this subcategory are Smithies’ (2011) rational-access view^13^ and Koralus’ (2014) erotetic (question-related) theory of attention.^14^

## Mechanistic definitions

Whereas *Behaviorist* [1] definitions rely on outward markers and *Phenomenal* [2] definitions of the character of experience, *Mechanistic* [3] definitions rely on the mechanisms subserving attention. Using Bechtel’s (2008, pp. 13–17) model of mental mechanisms, *Mechanistic* definitions identify attention by the *Parts* [3.A] in the brain that subserve it, the *Operations* [3.B] those parts perform, and the ways that those parts and operations are *Organized* [3.C]. *Operational* definitions are perhaps the most common in the world of cognitive science today.

### Parts

Attention might be defined as activity in the brain structures that subserve it. But there are at least two ways to delineate these parts: *Anatomical Regions* [3.A.I] and *Connectomes* [3.A.II].^15^

#### Anatomical Regions

There has been some progress made on identifying brain regions that are involved in specific types of attention. For example, the fronto-parietal regions have been implicated in fMRI studies with top-down visual, auditory, and tactile attention signals, while bottom-up attention seems to be subserved by localized modality-specific regions, such as V1 for visual saliency (Kanwisher & Wojciulik, 2000). But few have been tempted to simply identify activity in a specific anatomical locus with attention, or to hold that without said activity attention must necessarily be absent. Attention is not localized in the brain: “Scientific research suggests that the class of such subpersonal attentional processes is large, highly diverse, and not well localized in the brain” (Watzl, 2011a, p. 163).

There is good reason to think that the vast majority of cognitive processes are related to brain areas in a many-to-many relation (Anderson, 2010; Anderson et al., 2013)—each function requiring many areas, and each area subserving many functions. Attention involves many brain regions and overlaps with the footprint of many other processes (Anderson et al., 2013; Naghavi & Nyberg, 2005; Rosenberg et al., 2017). What is more, very similar acts of attention, such as task switching, may involve very different parts of the brain (Wager et al., 2005). Given all this, an anatomically circumscribed localized “attention center” is highly implausible.^16^

#### Connectomes

An alternative to the *Anatomical Regions* approach might be a network approach that carves up the brain according to *Connectomes* of neurons that communicate heavily with each other, even though they are distributed throughout many anatomical regions of the brain. Mogensen and Overgaard’s (2018) *Reorganization of Elementary Functions* framework is a promising account that respects the many-to-many relationship and accounts for both functional localization and the apparently contradictory capacity of brains for recovering abilities after trauma by conscripting completely different anatomical and neural structures to perform the lost functions. Thus, there are both anatomically localized functional units—“elementary functions”—and long-range connections between these units. Such an account is more felicitous given that attention of some kind likely permeates most cognitive processes.

*Connectomes* snaking through large swathes of the brain are virtually impossible to lesion in isolation from their surrounding neurons—whether experimentally or by disease or injury—even if we could identify them in a subject in vivo. And while we have imaging tools of reasonable sensitivity to explore the activity of *Anatomical Regions*—fMRI for example—we lack satisfactory tools for selectively measuring connectome activity in vivo. EEG (Eimer, 2015) and MEG (Baillet, 2017) can give some information, but it is of frustratingly low resolution. What is more, there remain serious problems with interpreting scans (Carp, 2012) and explanatorily bridging neural activity with cognitive functions generally (Fine, 2010, pp. 281–282; Naselaris et al., 2018, p. 3).

So, defining attention by the parts of the brain involved seems to be of limited utility, not only because attention is so widespread in brain activity, but also because of our investigative limitations. However, considering brain operations is more promising.

### Operations

In cognitive science, attention is usually defined in terms of its functional role, rather than its phenomenology. (Smithies, 2011, p. 250)

The *Operations* [3.B] of the brain can profitably be viewed as functional roles.^17^ The challenge here is to distil those functional roles that are specifically best considered as attention, rather than something else (e.g., *storage* or *binding*, see Sect. 4).

#### Access

Attention may be defined as simple *Access* to data or content, or variations thereof (*Abundance, Increase, Amplification, Maintenance, Influence, Effects*), but not, I argue, mere *accessibility*. On the view that attention is just simple *Access* [3.B.I.a], whatever is being processed is therefore being attended. This approach suffers the serious drawback of making attention merely synonymous with cognition, and therefore a superfluous term, so it is not surprising that it finds few proponents in modern discourse. The subjects undeniably have some sort of *Access* to the gorilla—its image falls upon the retina and must therefore register in the earliest stages of visual processing—yet they clearly do not attend to it at all.

A more useful approach is one which employs the concept of a relative *Abundance* [3.B.I.b] of some cognitive quantity. A process that recruits more cognitive processing power is attended while one that recruits less is not. Perhaps there may be a threshold,^18^ above which a process is deemed to be attended, or it may be a graded affair of greater or lesser degrees of attention. Subjects do not attend to the gorilla because they devote few cognitive resources to perceiving and/or reporting the gorilla. Thus also, Kanai et al., (2006, pp. 2334–2335) correlate spatial attention with abundant activity of the orientation-perceiving circuits relating to that location in a visual field.

*Increase* [3.B.I.c] captures the idea of recruiting *more* cognitive processing resources over time. Thus, the gorilla is not attended unless its retinal image in the early stages of visual processing is passed on to later stages, or passes beyond a certain stage of processing, or exceeds some threshold of processing resource use. *Amplification* of input signals (Fazekas & Nanay, 2018) is a neural version of increase.

The thing that is *Abundant* or being *Increased* need not be just “processing” generally, but may be defined more narrowly as a certain kind of processing. For example, attention may be the increase of “access to conscious experience” (Baars, 1988, p. 302), or to particular contents of conscious experience or unconscious content.

One way to facilitate increased access or processing is the *Maintenance* [3.B.I.d] of *Access* over periods of time (Kane & Engle, 2002), e.g., the reinforcement of a trace in working memory so that it might persist longer and thus continue to be *Accessed* by other cognitive processes.

Of course, *Maintained* or *Increased Access* or cognitive processing implies that the outputs of attended processes will also be more *Influential* or have a greater *Effect* [3.B.I.e] on the cognitive economy. This idea is at the heart of biasing accounts of attention (Desimone & Duncan, 1995), and it has been suggested that attention influences the character of perceptions (Carrasco et al., 2004; Carrasco & Barbot, 2019; Ling, 2012) and interactively influences motor movements (Moore et al., 2003).^19^

There is a distinction to be drawn between actual *Access* and merely potential *accessibility* or *availability* (Chalmers, 1997; Dehaene et al., 2006). But it is hard to see how content that is *potentially* directly available but not *actually Accessed* is in any helpful sense thereby attended. Surely the gorilla was always potentially accessible, but we consider it unattended precisely because it is never actually *Accessed*.

#### Detection and Recognition

Another approach focuses on the idea of *Detection* or *Recognition* [3.B.II]. Attention is the process of “detecting signals^20^ for (conscious) processing” (Posner & Petersen, 1990, p. 26). This process of detection can itself be a complex one, and attention may operate in any of its stages. These include: *Orienting*; *Vigilance or Monitoring*; *Scanning or Searching*; *Expectation*; *Alerting*; and *Tracking*.

*Orienting* [3.B.II.a] is the act of rearranging one’s physical deportment in space to better receive information from the environment. Classical examples of this are turning one’s head to see better, and rapid saccades of the eyes. Petersen & Posner (2012) consider orienting to be one of three defining characteristics of attention, together with alerting and executive control. But Prinz considers orientation and attention to play two distinct roles: “Informally, orienting alters what information gets in, and attention alters where it flows” (Prinz, 2011, pp. 193–194). Nonetheless, the act of *Orienting* itself—whether physically moving body parts or even the shifting of the focus of spatial attention while remaining physically still—does seem to constitute an integral part of some kinds of attention. The act of *Orienting* the eyes towards the ball is surely an integral aspect of a subject attending its changing position, while the lack of such *Orientation* is integral to the lack of attention to the gorilla.

*Vigilance* or *Monitoring* [3.B.II.b] is non-specific receptiveness to *any* new incoming content (contrasted with *Searching* for a specific target, below). The gorilla might have been attended by subjects had they been asked to simply attend to and report anything at all they saw, rather than count the number of passes. This state of openness has been called a kind of “preparatory attention” (Zeman, 2001, p. 1274).

The pass counting task itself was an example of the more narrowly specified *Scanning or Searching* [3.B.II.c]—the goal-directed search for something in particular. This is James’ (1890, p. 434) idea that one of the functions of attention is to hold in the mind an image of the thing one is searching for, so that one can identify that thing by comparison to the image.

Closely related to *Monitoring* and *Scanning* is the concept of *Expectation* [3.B.II.d], where top-down influences shape the course of attention, as, for example, in perceptual priming (Naccache et al., 2002). Like *Scanning or Searching*, *Expectation* might be considered a form of the more general *Vigilance or Monitoring* but with a more specific object/s. Unlike *Scanning or Searching*, it often occurs subconsciously. The instructions given to the subjects lead them to *Expect* only players and a basketball—gorillas are unexpected, and therefore, not attended.

The act of finding the object of the search is what Petersen and Posner (2012) call *Alerting* [3.B.II.e]—the signal that something has actually been detected or recognized. This *Alerting* is absent in the case of subjects who fail to see the gorilla, and reliably indicates the absence of attention to the gorilla.

*Tracking* [3.B.II.f] is what happens when one has found a target, and then maintains focus on that target over a period of time.^21^ The subjects identified the basketball, attended to it due to a top-down imperative, and maintained that attention as the ball was passed from player to player. O’Regan and Noë (2001, p. 944) suggest that tracking a target while otherwise occupied is the signature of moving from inattention to that target, to attention. One may *Track* multiple moving targets of attention simultaneously (Franconeri et al., 2007, p. 1011).

#### Selection

Our cognitive economy is engulfed constantly in a raging flood of information, whether exteroceptively from outside, via sensory perception, or interoceptively from within. How can the cognitive economy deal efficiently with all this information and tame it into James’ efficient stream of consciousness? How can it sift the relevant from the irrelevant, and choose to act accordingly? One of the most prominent definitional approaches to attention views it as being primarily *Selection*^22^ [3.B.III] that facilitates the efficient management of this constant flood of information. In the mid twentieth century, the *bottleneck* model of attention enjoyed a vogue, where attention was seen as a unitary mechanism of constriction of processing capacity at some stage in the chain of cognitive processing (Broadbent, 1958, 1971). This led to the question of whether such selection occurs early or late in the processing chain (Pashler, 1998, pp. 13–19). More recently, Baars (1997, p. 368) characterizes attention as *selection* in contrast to consciousness as *experience*, while Campbell (2011) contrasts *selection* with *access*. Here, I catalogue a variety of ways in which the general idea of selection has been applied to the definition of attention: *Filtering*; *Gateway* metaphors; *Spotlight* metaphors; *Zooming In/Out*; and the ideas of *Exclusion*, *Competition*, and *Selection for Action*.

At the heart of the bottleneck model is the idea of *Filtering* [3.B.III.a] (Broadbent, 1958)—sifting out a small, manageable, and relevant amount of content from a larger unruly heap (the gorilla, being irrelevant to the present task of counting passes, was filtered out of the subject’s focus). The nature of this *Filtering* is complex (Baars, 1988, pp. 34–36). The *frame problem* in artificial intelligence^23^ can be seen as being solved in human cognition through attention-as-filtering.

Attention has been defined by what it selects *for*—the metaphor of a *Gateway* [3.B.III.b] leading to something else (further down the processing chain). Examples of attention-as-gateway include the *Gateway* to: visual processing (Desimone & Moran, 1985); consciousness (Baars, 1988, p. 369; Crick & Koch 1990, p. 269; Mack & Rock 1998, p. 25); and working memory (Awh et al., 2006, p. 202; de Brigard & Prinz 2010, p. 52). Of course, these *Gateways* are not mutually exclusive—any number of destinations may lie on the other side of the gate. Attention is the *Gateway* to reporting either passes or gorillas.

*Spotlight* [3.B.III.c] theories of attention (Eriksen & Hoffman, 1972) emphasize the idea that what determines whether or not attention is being paid to a stimulus is its location (Mole, 2017, Sect. 2.7). While spatial location seems to be a major factor in how attention is directed generally, there are good reasons for holding that it is certainly not the *only* factor. For example, one might argue that the gorilla undoubtedly passed through the moving spatial *Spotlight* of the subjects’ attention when the ball passed directly in front or behind it, yet it still failed to be attended. Pure *Spotlight* theories cannot account for pop-out in visual search where what brings the stimulus to attention is its contrast against a background, not its spatial location (Wolfe, 1994). Neither can spatial attention explain olfactory attention, or much of auditory attention, which are not spatial by nature.^24^

The metaphor of a *Spotlight* naturally raises the question of the dimensions of that *Spotlight*. How narrow or wide is its beam? And is it possible to vary its dimensions, to “*Zoom In/Out*” [3.B.III.d] as it were (Eriksen & St James, 1986)? The gorilla is not attended because the *Spotlight* has been *Zoomed In* very tightly upon the ball. Should a subject *Zoom Out*, the gorilla might well come into attention.

*Selection* involves the inclusion of some content, which therefore means that other content is *Excluded* [3.B.III.e]. When one selects the basketball, one is automatically thereby not selecting (*Excluding*) the items left over, including the gorilla. This approach to defining attention may be traced back at least to JS Mill: “The expression [attention] means that a sensation tends more or less strongly to exclude from consciousness all other sensations” (cited in de Brigard & Prinz, 2010, p. 52), and Treisman (2003, p. 102) contrasts the metaphor of an actively excluding attentional *window* with that of an inclusive attentional spotlight. Thus, *Exclusion* may occur via *Suppression*. van Swinderen (2005, pp. 327–328) suggests that in both sleep and alert attention (as opposed to being in an alert yet inattentive state), we raise the threshold required by peripheral inputs to enter into consciousness.

The intense concentration on the attended target to the *Exclusion* of all other signals may be described as *Absorption* (Tellegen & Waller, 2008). An extreme case may be savants like Kim Peek, where a *Suppression* of distracting stimuli seems responsible for their superhuman powers (Treffert & Christensen, 2005). Related to *Absorption* are the total *Dedication* of available resources to a task at the expense of other tasks (Mole, 2011b, p. 67) and the idea of *Absence of Distraction* (e.g., Cowan & Morey, 2006; Engle et al., 1999, p. 104).

One way that *Selection* of cognitive content can occur is via a process of cognitive *Competition* [3.B.III.f]. In the *Competition* for cognitive resources, the basketball wins hands down over the gorilla, due to the top-down imperative to focus on counting passes. Mole (2017, Sect. 2.6) considers *Competition* models to be the “clearest non-bottleneck mechanisms for achieving selectivity,” and discusses the sub-varieties and nuances of competition models. One of the most influential of these is *biased competition* (Desimone & Duncan, 1995; Ruff, 2011), which introduces the idea that other processes in the brain, whether top-down, bottom-up, or lateral, interact with the process of attention to bias its selection “choices.” This kind of approach has also been framed in predictive processing terms (Clark, 2016, pp. 59–63). Some, however, have sought to distinguish *Competition* from attention, arguing that “attention doesn’t refer to competition, as such, but, rather to a process that occurs when a competition is won” (Prinz, 2011, p. 183).

Finally, recognizing that attention is not restricted to perception is the idea of attention as *Selection for Action* [3.B.III.g] (Allport, 1987; Treisman, 1969; Wu, 2011, 2016). “Action” is used here in the more general sense that encompasses both mental and physical events, whether in the brain or the body, whether actually performed or merely mentally rehearsed or intended, so this definition is closely related to attention-as-influence (3.B.I.e). Subjects *Selectively* attended only to content relevant to the *Action* of counting passes. Others have also seen an intimate connection, perhaps even a unity of mechanism, between attention, working memory and motor action (Postle, 2006; Theeuwes et al., 2005).

#### Control

Human goals tend to be complex and extended over time. Coordinating the cognitive processes required to sustain the pursuit of such goals is therefore a complex task. When the subjects set out to count the number of passes, one might argue that they could not achieve this goal without attention-as-*Control* [3.B.IV]. Parasuraman (1998, pp. 7–8) points out that such mechanisms of attentional control do indeed feature heavily in executive and planning components (Norman & Shallice, 1986) of popular models of working memory (Miyake & Shah, 1999). Attention can therefore be thought of as the mechanism that directs the cognitive traffic in a cognitive economy in order to ensure the smooth and most effective and efficient functioning of that economy in its constant striving to fulfil those complex goals.

#### Coherence

If attention serves to make content more manageable, one of the ways it might achieve this is by making it *Coherent* [3.B.V]. On these accounts, attention may be defined by its functional role in separating out and coordinating a limited, relevant, and coherent body of information as the foundation for further processing in things like reasoning, agency, and action. The sensory data from the basketball passes is processed to form a *Coherent* story about the movement of the ball from player to player in the accepted context of a basketball game (of sorts). The trespassing gorilla is *incoherent*—difficult to reconcile with this context, and so is ignored, i.e., unattended. Attention-as-*Coherence* is involved in the *Feature Integration Theory* (Treisman, 2003; Treisman & Gelade, 1980), where attention helps bind disparate features into *Coherent* perceptual wholes. In predictive coding models, attention is the process of optimizing precision of signals, a kind of *Coherence* (Friston, 2009; Hohwy, 2012). Mole’s (2011a, 2011b) *cognitive unison view* also plausibly falls under this subcategory. In a similar vein, Wyble (2015) creatively describes visual attention as being not so much a filter or a newspaper editor, picking which stories to publish, but a movie editor, arranging scenes into a *Coherent* story.

### Organization

The final category of Bechtel’s framework focuses not on which *Parts* of the brain are involved in attention, nor on what those parts do, but on how the *Parts* are organized in relation to each other. In relation to attention, this *Organization* [3.C] can be examined on many levels: the relationships of individual neurons to each other; of networks of neurons to each other, of *Anatomical Regions* [3.A.I] to each other, or of functionally specific *Connectomes* [3.A.II] to each other. Alternatively, we could look at organization at Marr’s (1982) three levels: computational theory, representation and algorithm, and implementation. *Organization* is not merely spatial but also temporal. Of course, these are not mutually exclusive accounts, but complement each other. A thorough analysis of attentional *Organization* is far beyond the scope of this paper, so I will just offer some brief remarks.

In what sense might *Organization* define attention? Bechtel’s triad of parts, operations, and organization are meant to be intimately connected. There is some hope that we might one day be able to identify the *Organization* of the various subtypes of attention, and how they relate to each other, whether neuronally or functionally. For example, Rosenberg et al. (2017, pp. 299, Box 1), adopting an individual differences approach, found that some of the functions subsumed under the umbrella term *attention* vary among individuals together, whereas others vary independently of each other. The ability to sustain attention over long periods of time does not necessarily covary with the ability to multitask. Spatial orienting, attentional capture, and inhibition of return seem to vary independently of other sub-functions. On the other hand, functions like search, tracking, and visual short-term memory all seem to vary together across individuals and over time, suggesting they may depend on some kind of common attention factor, at least in part. These patterns of co-variance might indicate underlying organizational patterns.

It will come as no surprise that the *Organization* characteristic of attention is complex. Attentional *Operations* require content to relate to other content, and processes to influence other processes. We should expect them, therefore, to be characterized by organizational patterns such as *Cyclic Pathways* [3.C.I] and *Feedback Loops* [3.C.II] (Bechtel, 2008, p. 17). What is more, parts and operations involved in attention will be intimately entwined—richly *Contextually Interconnected* [3.C.III]—with other cognitive systems, contentfully, functionally, and neuronally. This has led some to identify *Organizational* neural structures that allow for recurrent neural firing patterns with attention (Ruff, 2011, pp. 8–9), although others have identified them with consciousness (Lamme, 2010). Like the *Parts* [3.B] subdefinitions of attention, *Organizational* subdefinitions have tended to be less prominent in the literature than *Operational* ones. This is a category that could be much improved and refined by those in the field of neuroscience.

## Using the taxonomy

The taxonomy of attention presents many opportunities for furthering our inquiry into attention in all sorts of ways—both conceptually and empirically—too many to attempt an adequate overview in this brief section. Here, I gesture towards just two possible lines of inquiry: is the umbrella term *attention* still useful?; and what is it that ties the subdefinitions of attention together yet distinguishes them from other kinds of cognition?

The taxonomy offers the possibility of dispensing with the umbrella term, *attention*, altogether, and using only the more specific terms that are its entries. This would certainly be a step in the direction of clearer, more precise definition in, say, empirical research. But while the more specific terms are certainly useful in many circumstances, the intuition that there is something important that ties them together is hard to shake, much as it is in the taxonomy of life or the dictionary.

Perhaps *attention* in cognitive science is something akin to the term *shopping* in economics. Both terms are very broad, cover a multitude of sub-varieties with significant overlap between them, and which can be taxonomized in various ways. Shopping can be online or in person; wholesale or retail; involve a huge array of providers, products (including both goods and services), consumers, and strategies for connecting them; and so on. And yet, as unmanageably broad as the term *shopping* is, it plays a very useful role in tying together this vast array of often disparate concepts in helpful and fruitful ways.

As a conceptual exercise, then, *attention* retains its value as the umbrella under which sometimes apparently disparate concepts are kept together in our thinking, providing a more solid context for inquiry into any one or any group of them. This principle is even more important in empirical research where the aim is to build an overall picture of how cognition works. Such a picture requires that we get the relations right, both among the subvarieties that fall under the umbrella term of *attention* and between attention (and its subvarieties) and other kinds of cognition. The taxonomy specifies a structure upon which such relations can be traced out.

This suggests another reason for retaining the term *attention*. One can make the case that when the subvarieties are considered, they can all be characterized by certain features that distinguish them from any other kind of cognition, thus suggesting that *attention* might be something of a natural kind.

The question then becomes, what is it that unites all the subvarieties of attention, but excludes all other kinds of cognition? Now, a number of proposals have been made as to what kind of thing attention might be: a system (Posner, 1994, p. 7399); a process (Kentridge & Heywood, 2001); a variable (Baars, 1997); or an adverb (Mole, 2011a). But perhaps the proposal that jumps out as being the one that best captures the unifying principle of the taxonomy is Watzl’s proposal that attention is the *structuring* of cognition (Watzl, 2010, 2011a). I propose that the uniting principle that ties the entries in the taxonomy together but excludes other kinds of cognition is that ***attention is a suite of strategies for selectively structuring cognition for further processing***.

A “strategy” account of attention most comfortably captures the *Mechanistic* [3] categories of the taxonomy. *Operations* [3.B] are the implementations of strategies. Providing the appropriate *Parts* [3 A] and *Organization* [3.C] are strategies for creating the conditions that allow such implementations. *Phenomenal* [2] attentional strategies are operations that have some particular kind of phenomenal experience as part of their essence (e.g., the phenomenal sense of *salience* [2.D]). And *Behavioral* [1] attentional strategies are the outward manifestations of the implementation of these strategies. These strategies are multiply realizable (Bickle, 2016)—even security cameras suitably equipped with servo motors can *monitor* [3.B.II.b], *orient* [3.B.II.a], and *track* [3.B.II.f]. And the apparent lack of an “attention centre” in the brain suggests that attentional strategies are implemented throughout cognition (Watzl, 2011b, p. 847); they form a component or aspect of many, perhaps most kinds of cognition. Further, a “strategy” account avoids the problems inherent in reductionist accounts of attention (Watzl, 2011b, pp. 846–848).

Attention-as-strategies is thus intimately related to other cognitive strategies, systems, and processes that implement those strategies, which may therefore be said adverbially to be attentional just when they do.^25^ Nonetheless, the principle above serves to dissect out that which is attention from that which is not. For example, *storage* (in memory) is conceptually dissectible from *attention*. The strategy of encoding content in a retrievable way requires *Selection* [3.B.III] of that content *for* encoding, but encoding is not itself selection. The essence of storage is that idea of temporal endurance, not *Selection*. Retrieval of a memory again requires the attentional strategies of *Access* [3.B.I], *Searching* [3.B.II.c], and *Alerting* [3.B.II.e] when the required memory has been found, but the essence of retrieval is the actual use of the recovered memory and therefore constitutes “further processing.” In a similar way, the essence of *binding* (of disparate data into whole perceptions or concepts) is not *Selection*, but the combination of content in principled ways.^26^*Coherence* [3.B.V] is a strategy implemented to help achieve that *binding* in useful ways, but is conceptually dissectible from the combining itself. Similar dissections can be made for other strategies such as *calculation*, *comparison*, *sensation*, and many more. This formulation of attention, therefore, drawn from the taxonomy, not only encompasses all the subvarieties in the taxonomy—it also provides the principle by which other cognitive strategies may be excluded from the taxonomy, and thereby, from being considered forms of *attention*.

These are just brief sketches of two possible ways that the taxonomy might be fruitfully employed, but there are many more. Empirical researchers can use it to quickly and clearly identify precisely which aspect of attention is the subject of their work, and to therefore situate it in relation to the work of others, even those outside their discipline. Studying the structure of the taxonomy might lead to insights into how the different entries and categories relate to each other, as is the case for both the dictionary and the tree of life, and therefore yield insights into the nature of each, and perhaps even into the nature of attention as a whole. On a broader scale, attention has been the subject of much inquiry that seeks to understand its relations to non-attentional phenomena such as consciousness, action, virtue, etc. (Mole, 2021, Sects. 3–4). A taxonomy of attention allows us to delineate precisely which subvarieties of attention are in play in any given discussion of this nature, and may highlight possible avenues for fruitful research into connections with hitherto unexplored subvarieties in that context. And so on.

## Conclusion

A taxonomy of attention—like a dictionary or a tree of life—would be a valuable tool to anyone interested in better understanding this multifaceted phenomenon. It is somewhat surprising that no serious attempt at a comprehensive taxonomy seems to have yet been attempted. The taxonomy I propose in this paper is a first draft of such a comprehensive taxonomy, and one that can be improved in future iterations. Like the different kinds of dictionaries (alphabetical, thesaurus, rhyming) there are undoubtedly other ways to structure a taxonomy of attention. Perhaps each of them would have its own advantages and disadvantages. But we may hope that developing and employing taxonomies of attention will serve to impel inquiry into attention in new and interesting directions, as have taxonomies in appropriately similar fields of inquiry. In Sect. 4, I provided the merest of hints of possible benefits of employing the taxonomy in inquiry into the phenomenon of attention. Not only might each of them be developed much further, but there are many other directions of inquiry that might benefit from employing the taxonomy.

We have reason, therefore, to hope that despite the unruliness of the term *attention*, a good taxonomy can master it and harness its potential, significantly improving its productivity. Humpty Dumpty would be proud.

## Data Availability

Not Applicable.

## Footnotes

1 Chapter XI, p. 403. This oft-quoted catchphrase reflects Munsell’s (1873, p. 11) less pithy but earlier statement: “On attention itself, it is needless to discourse at length; its nature and conditions are familiar to every thoughtful student.” Hommel et al. (2019) present a more nuanced view than their pithy title might suggest.

2 Some taxonomic work is extant taxonomy (Arvidson, 2003; Chun et al., 2011; Mole, 2011b, pp. 62–63; Regan et al., 2011; Rensink, 2015; Treisman, 1969) but none of these is as comprehensive as the present taxonomy. For a brief analysis of existing taxonomic work, see Kaldas (2019, Sect. 3.2.4.4).

3 My approach also meets JH Taylor’s (2015, pp. 40–41) two criteria for defining attention. It is *extensionally adequate* in that it allows for varying degrees of “leniency” in defining attention, depending on the needs of the task at hand. It is *non-circular* in that it is based on a descriptive approach rather than a stipulative approach.

4 They certainly apply to non-human living creatures (animals, insects, etc.) Whether they may be applied to groups of people or animals (social cognition), or even to non-living systems (e.g., artificial intelligence), are interesting questions.

5 See also a discussion of his view, and comparison with those of Alexander Bain, in Mole (2017, Sect. 1.5).

6 The question of whether attention is necessary and sufficient for phenomenal consciousness is an open one; see Kaldas (2019).

7 For a summary of some of these subcategories of Phenomenal Attention, including citations to Early Modern and Modern examples, see Watzl (2011b, p. 843).

8 On the other hand, Matthews et al. (2018, p. 5) found that subjective confidence ratings track objective *accuracy* rather than attention.

9 For an argument against the suggestion that vividness can vary independent of content, see Bourget (2017).

10 Stazicker (2011, p. 172, footnote 12) describes Leibniz’s ideas of clarity and vividness in relation to attention and consciousness. Phillips (2011, p. 221, note 4) observes that discussions of attention as “clearness” may be found in authors such as Titchener, Woodward, and Gill and Dallenbach in the early twentieth century. A modern revival of the idea is Jennings (2012).

11 For a discussion of the different roles played by salience and attention in this dance in the context of making moral decisions, see Chappell and Yetter-Chappell (2016, p. 454). For a discussion of the neural level at which salience operates in the mechanism of attention, see Fellrath and Ptak (2015) and Parkhurst et al. (2002), and for a predictive processing account, see Clark (2016, pp. 28, 66–69).

12 Jennings (2015, pp. 288–289) discusses an interesting development of this approach in which she speaks of “focus without the aid of attention”.

13 “On this view, attention is a distinctive mode of consciousness … it makes information accessible for use in the rational control of thought and action.” (pp. 247–248). Were it not for the characterization of attention as a “mode of consciousness,” Smithies’ view might have fitted better under *Selection for Action* (3.B.III.g) or *Control* (3.B.IV).

14 “Erotetic” means relating to questions. Thus, “On the erotetic theory, the functional role of attention is a matter of the relationship between questions and what counts as answers to those questions … The erotetic theory proposes an integrated account of attention in cognitive control and of attentional focus in perception. The functional role of attentional focus on objects, properties, and locations has to do with picking out something that corresponds to what a task is ‘about’.” (p. 26).

15 Much less likely ways are by type of neurone, or by predominant neurotransmitter (e.g., Schmitz & Duncan, 2018).

16 Naghavi and Nyberg (2005) review a number of empirical studies that found correlations between specific brain regions and specific kinds of attention. They also found that there are areas of the fronto-parietal regions that seem increase their activity during all four of attention, conscious visual perception, working memory, and episodic memory retrieval. The significance of such findings continues to be debated.

17 For a similar, though less detailed or structured list, see Ganeri (2017, p. 224).

18 See Dehaene et al. (2006) for a similar threshold model relating to consciousness rather than attention.

19 See also attention-as-selection-for-action (3.B.III.g).

20 I imagine the signals may be sensory inputs or abstract thoughts, emotions, beliefs, etc.

21 For an evolutionary account of the tools humans use to make their tracking more effective, see Menary (2018).

22 “The selective nature of attention has perhaps been the most widely studied area” (Rees, 1999).

23 The frame problem first arose as a technical problem in artificial intelligence (McCarthy & Hayes, 1969) where it has largely been resolved today. Roughly, it is the problem about how to know what to sift out from the flood of data available in a real-world environment so that only what is relevant to the task at hand is focused on. It continues to be debated and explored in cognitive science (Dennett, 2006; Shanahan, 2016).

24 Keller (2011) argues that the current focus on visual attention at the expense of other modalities risks obscuring important aspects of attention.

25 This is why I rejected “accessibility” as an attentional definition above (Sect. 3). We can apply the adverb “attended” iff one of the attention strategies is actually being implemented, not if it is only potentially implementable.

26 *Pace* Treisman and Gelade (1980).
